# Cooking-Induced Oxidation and Structural Changes in Chicken Protein: Their Impact on In Vitro Gastrointestinal Digestion and Intestinal Flora Fermentation Characteristics

**DOI:** 10.3390/foods12234322

**Published:** 2023-11-29

**Authors:** Guanhua Lv, Hengpeng Wang, Xiaoou Wei, Minmin Lu, Wenhao Yang, Halah Aalim, Esra Capanoglu, Xiaobo Zou, Maurizio Battino, Di Zhang

**Affiliations:** 1 School of Food and Biological Engineering, Jiangsu University, Zhenjiang 212013, China; 2212118055@stmail.ujs.edu.cn (G.L.); yzuwhp@163.com (H.W.); xiaoou_wei1997@163.com (X.W.); 2212118064@stmail.ujs.edu.cn (M.L.); ywh13615265085@163.com (W.Y.); a.halah@outlook.com (H.A.); d.zhang@ujs.edu.cn (D.Z.); 2 Key Laboratory of Chinese Cuisine Intangible Cultural Heritage Technology Inheritance, Ministry of Culture and Tourism, College of Tourism and Culinary Science, Yangzhou University, Yangzhou 225127, China; 3 Department of Food Engineering, Faculty of Chemical and Metallurgical Engineering, Istanbul Technical University, 34469 Istanbul, Türkiye; capanogl@itu.edu.tr; 4 International Joint Research Laboratory of Intelligent Agriculture and Agri-Products Processing, Jiangsu University, Zhenjiang 212013, China; 5 International Research Center for Food Nutrition and Safety, Jiangsu University, Zhenjiang 212013, China; 6 Department of Clinical Sciences, Faculty of Medicine, Polytechnic University of Marche, 60121 Ancona, Italy

**Keywords:** chicken, cooking method, protein oxidation, in vitro simulated digestion, intestinal flora, short-chain fatty acids

## Abstract

Meat digestion and intestinal flora fermentation characteristics are closely related to human dietary health. The present study investigated the effect of different cooking treatments, including boiling, roasting, microwaving, stir-frying, and deep-frying, on the oxidation of chicken protein as well as its structural and digestion characteristics. The results revealed that deep-fried and roasted chicken exhibited a relatively higher degree of protein oxidation, while that of boiled chicken was the lowest (*p* < 0.05). Both stir-frying and deep-frying led to a greater conversion of the α-helix structure of chicken protein into a β-sheet structure and resulted in lower protein gastrointestinal digestibility (*p* < 0.05), whereas roasted chicken exhibited moderate digestibility. Further, the impact of residual undigested chicken protein on the intestinal flora fermentation was assessed. During the fermentation process, roasted chicken generated the highest number of new intestinal flora species (49 species), exhibiting the highest Chao 1 index (356.20) and a relatively low Simpson index (0.88). Its relative abundance of *Fusobacterium* was the highest (33.33%), while the total production of six short-chain fatty acids was the lowest (50.76 mM). Although stir-fried and deep-fried chicken exhibited lower digestibility, their adverse impact on intestinal flora was not greater than that of roasted chicken. Therefore, roasting is the least recommended method for the daily cooking of chicken. The present work provides practical advice for choosing cooking methods for chicken in daily life, which is useful for human dietary health.

## 1. Introduction

Chicken, as one of the most commonly consumed poultry meats, possesses a rich nutritional profile, including essential amino acids, unsaturated fatty acids, and other micronutrients [1]. It is renowned for its tender texture, delicious taste, and widespread popularity worldwide [2]. Furthermore, studies have indicated that poultry consumption is less likely to cause cardiovascular diseases compared with red meat [3]. Therefore, chicken, as the representative poultry meat, may be a healthier choice for meat consumers.

Cooking plays a vital role in transforming raw meat into a palatable form, and there are various cooking methods for different types of meat [4]. However, during the cooking process, meat may undergo the oxidation of proteins and lipids, along with changes in protein conformation [5]. These changes can subsequently affect the digestion of meat in the human body [6]. A previous study reported that heating significantly increased the carbonyl content of steaks and reduced their in vitro digestibility [7]. Conversely, it has been demonstrated that mild heating conditions increased the digestibility of pork protein [8]. Interestingly, it has been discovered that a shorter cooking time improved the in vitro digestibility of bovine collagen, whereas a longer cooking time reduced its binding ability to pepsin and thus its overall digestibility [9]. While these studies have provided some insights into the relationship between cooking intensity and the digestion characteristics of meat, they often focus too much on the effects of varying time and temperature within a specific cooking method or rely on processing methods used for commercially available meat products. Currently, there are a limited number of studies concerning the digestion characteristics of meat under different common home cooking methods. This is necessary, as it is more closely related to the daily dietary habits and overall health of people.

What is more noteworthy is that residual undigested proteins during gastrointestinal digestion can reach the colon and subsequently influence the growth of the intestinal flora [10]. The amount of protein reaching the colon depends on protein intake and digestibility [11]. It was reported that a high-protein diet is more likely to cause ulcerative colitis and Crohn’s disease [12]. Another study has shown that a low-protein diet significantly reduces protein fermentation products and increases the number of beneficial bacteria in the gut [13]. These studies suggest that changes in the digestion characteristics of meat may show diverse effects on the intestinal flora, which are closely linked to human dietary health [14]. Unfortunately, most studies have overlooked the impact of changes in meat digestion characteristics caused by different cooking methods on the intestinal flora.

Thus, we conducted a study using chicken as a model to investigate the changes in the oxidation, structure, and digestion characteristics of protein resulting from five common home cooking methods (boiling, roasting, microwaving, stir-frying, and deep-frying). Further, we explored how these changes affect the growth of intestinal flora during colonic fermentation. Our findings aim to provide practical recommendations for the selection of cooking methods in daily life, which holds significant implications for human dietary health.

## 2. Materials and Methods

### 2.1. Materials and Reagents

Fresh chicken breasts were procured from Jimailong Supermarket (Zhenjiang, China). The following chemicals and reagents were purchased from Sigma-Aldrich (Shanghai, China): α-amylase (14 U/mg), pepsin (3000 U/mg), pancreatin (USP level), bile salt (BR), 5,5’-Dithiobis-(2-nitrobenzoic acid) (DTNB), bromophenol blue (BPB), Tris-Glycine buffer, phosphoric acid and Nile blue. All reagents utilized in this study were of analytical grade.

### 2.2. Sample Pretreatment and Cooking Procedure

The defrosted chicken breasts were cut into similar-sized chunks (3 × 3 × 3 cm^3^). All of the chicken pieces were divided into six groups, five of which underwent different cooking treatments, namely boiling (BO), roasting (RS), microwaving (MW), stir-frying (SF), and deep-frying (DF), leaving a group of uncooked raw meat (R) as the control group. The detailed cooking procedures are shown in Table S1.

### 2.3. In Vitro Oral-Gastrointestinal Digestion and Colonic Fermentation

#### 2.3.1. In Vitro Oral-Gastrointestinal Digestion

The cooked chicken samples underwent in vitro digestion according to the method described previously [15,16] with some modifications. The specific components of the gastrointestinal simulated electrolyte solution and the specific scheme for in vitro simulated digestion are shown in Table S2 and Figure S1.

The gastric (G) and gastrointestinal (GI) digested samples were subjected to a 10 min incubation in a boiling water bath (DF-101S, Lichenbangxi Scientific Instrument Co., Shanghai, China) to halt the digestion process. Following this, the samples were centrifuged at 5000 rpm and 4 °C for 5 min (TG16-WS, Xiangli Scientific Instrument Co., Changsha, China). The supernatant was used to detect digestion characteristics, and the precipitates were freeze-dried (FD-1-50, BIOCOOL Instrument Co., Beijing, China) and stored at −80 °C as substrates for colonic fermentation.

#### 2.3.2. In Vitro Colonic Fermentation

The Simulator of the Human Intestinal Microbial Ecosystem (SHIME^®^) was used to study colonic fermentation in vitro, referring to the method described previously [17]. The fecal collection, the composition of the culture medium (Table S3) and the stability of the initial flora (Figure S2) are described in the Supplementary Materials.

### 2.4. Determination of Protein Oxidation

#### 2.4.1. Quantification of Free Sulfhydryl Content

The determination of free sulfhydryl content referred to the method reported [18] with some modifications. First, 2 g meat samples were homogenized in 10 mL of PBS (phosphate-buffered saline). After centrifugation (6000 rpm, 4 °C, 10 min), 0.5 mL of supernatant was obtained and 4.5 mL of Tris-Glycine buffer was added. Then, 0.5 mL of Ellman reagent was mixed homogenously with the sample and allowed to react at 25 °C for 30 min. Subsequently, the absorbance of the reaction mixture was measured at 412 nm using a U-3900 spectrophotometer (Hitachi Co., Tokyo, Japan).

#### 2.4.2. Quantification of Total Carbonyl Content

The detection of protein carbonyl was based on the principle that protein carbonyl reacts with DNPH (2,4-dinitrophenylhydrazine) to form 2,4-dinitrophenylhydrazone [19]. The content of protein carbonyl in chicken was determined using the carbonyl assay kit (Sangon Biotech Co., Ltd., Shanghai, China), and the carbonyl content was expressed as nmol per mg of protein.

#### 2.4.3. Determination of Schiff Bases (SB)

The determination of Schiff base content was performed using a modified version of the method described previously [20]. Samples were collected and measured for fluorescence intensity using a fluorescence spectrophotometer (F-2700, Hitachi Co., Tokyo, Japan). The emission spectrum ranged from 380 to 600 nm and the excitation wavelength was 360 nm. Both the excitation and emission slits were 5 nm. All measurements were repeated three times and the fluorescence intensity was expressed in arbitrary units.

### 2.5. Analysis of Protein Structure

#### 2.5.1. Determination of Protein Surface Hydrophobicity

According to the method described previously [21] with a slight modification, first, 1 mL of protein solution (1 mg/mL) was mixed with 80 µL of BPB solution, then the mixture was stirred for 10 min at room temperature using a magnetic stirrer (84-1A, Sile Instrument Co., Shanghai, China), followed by centrifugation at 7000 rpm and 4 °C for 15 min. The absorbance of the supernatant was measured at 595 nm with a U-3900 spectrophotometer (Hitachi Co., Tokyo, Japan). Meanwhile, 1 mL of 20 mM PBS was added to 80 μL of BPB solution as a blank group.

#### 2.5.2. Detection of the Secondary Structure of Protein

According to a modified version of the method described previously [22], a Fourier infrared spectrometer (Shimadzu Co., Kyoto, Japan) was utilized to collect spectral data. The scanning range was set from 4000 to 400 cm^−1^, with 64 scans performed at a resolution of 4 cm^−1^. The absorption spectra from 1700 to 1600 cm^−1^ were obtained for the analysis of the amide I band. Fourier automatic deconvolution and second derivative peak fitting were performed for spectral images using PeakFit (version 4.12, SPSS Inc., Chicago, IL, USA) software, and the relative contents of protein secondary structures were calculated.

### 2.6. Determination of In Vitro Protein Digestion Characteristics

#### 2.6.1. Determination of Protein Digestibility

The digestibility of chicken protein was determined by referring to the method described previously [23] with some modifications. After digestion, the supernatant was separated after centrifugation at 12,000 rpm for 10 min at 4 °C. The protein content in the supernatant was quantified using the Biuret method, and the digestibility of protein after digestion by the stomach and small intestine was calculated. The results were expressed as percentages (%).

#### 2.6.2. Sodium Dodecyl Sulfate Polyacrylamide Gel Electrophoresis (SDS-PAGE)

According to a previous study [24], the SDS-PAGE analysis of chicken in different cooking treatments before and after in vitro GI digestion was carried out using a 5% stacking gel and a 12% separating gel. In addition, the intensities of the chicken protein bands were measured using ImageJ (NIH, Bethesda, MD, USA).

#### 2.6.3. Microstructure

The microstructure of chicken treated using different cooking methods was observed via laser confocal microscopy (Deltavision OMX SR, GE Co., Piscataway, NJ, USA) before and after digestion, referring to a modified version of the method described previously [25]. First, 20 μL of 1% Nile blue was added to the 300 μL sample (digestive supernatant or protein extraction solution). The mixture was thoroughly shaken and then left to stand in a dark environment for a duration of 15 min. Then, the dyed sample was dropped onto a slide, and an image was obtained using a 40× objective lens and a 633 nm helium-neon laser.

### 2.7. Intestinal Flora Analysis of 16S rRNA Gene Sequencing

The intestinal flora analysis referenced the method described previously [10], with the specific details described in the Supplementary Materials.

### 2.8. Quantification of Short-Chain Fatty Acids (SCFAs)

The method reported previously [26] was used for the determination of SCFA content. In this process, 1.5 mL of fermentation supernatant was mixed with 0.1 mL of phosphoric acid (45%, *v*/*v*), and the mixture was injected into a gas chromatography (Shimazu Co., Kyoto, Japan). The injector temperature was 230 °C, and the detector temperature was maintained at 250 °C. In addition, the injection volume was 1 μL, and a shunt ratio of 1:10 was used.

### 2.9. Statistical Analyses

All experiments in this study were replicated at least three times. SPSS software (version 26, SPSS Inc., Chicago, IL, USA) was used for the analysis of variance (ANOVA) (*p* < 0.05). A comparison of the mean values was performed using the Tukey test. All images were created using Origin 2018 (Origin Lab Inc., Northampton, MA, USA).

## 3. Results and Discussion

### 3.1. Impact of Various Cooking Methods on the Degree of Chicken Protein Oxidation

#### 3.1.1. Free Sulfhydryl Content

**Figure 1:**
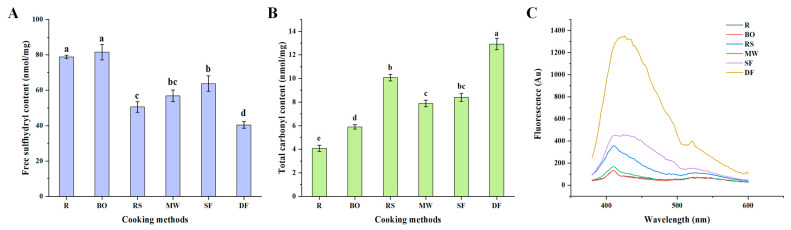
Effects of different cooking methods on chicken protein oxidation. (**A**) Free sulfhydryl content. (**B**) Total carbonyl content. (**C**) Schiff base content. R: uncooked raw meat. BO: boiling. RS: roasting. MW: microwaving. SF: stir-frying. DF: deep-frying. Different lowercase letters represent statistically significant differences between each group (*p* < 0.05).

The content of free sulfhydryl groups can be used to characterize the degree of protein oxidation. A higher degree of oxidation is associated with a greater loss of free sulfhydryl groups [27]. As seen in Figure 1A, compared with the R group (78.85 nmol/mg), only BO treatment (81.61 nmol/mg) showed little effect on the free sulfhydryl content, while the other four cooking treatments significantly reduced the contents of free sulfhydryl groups (*p* < 0.05). Among them, the DF treatment (40.42 nmol/mg) resulted in the greatest loss of free sulfhydryl groups, followed by the RS treatment (50.55 nmol/mg), suggesting that high temperatures might exacerbate protein oxidation. Conversely, milder cooking methods such as the BO and MW treatments exhibited a lesser effect on protein oxidation, which was consistent with a previous study [28]. Interestingly, the SF group (63.78 nmol/mg) exhibited a relatively smaller loss of free sulfhydryl content. This might be attributed to the absence of a stable heat transfer process during SF cooking.

#### 3.1.2. Total Carbonyl Content

Protein carbonylation is a non-specific and irreversible reaction that occurs in the early stages of the oxidation process. It is widely recognized as an important indicator for characterizing protein oxidation [29]. As depicted in Figure 1B, the carbonyl content of all samples significantly increased after cooking. Specifically, the SF and RS groups, respectively, exhibited around 1-fold and 1.5-fold increases compared to the R group. The DF treatment resulted in the highest carbonyl content (12.92 nmol/mg) (*p* < 0.05). This finding aligned with the effect of cooking treatments on the free sulfhydryl group content discussed in Section 3.1.1. A similar phenomenon was also reported in [30]. Our results demonstrated that heat treatment intensified protein oxidation, with the more intense cooking methods such as DF exhibiting a greater impact on the degree of protein oxidation.

#### 3.1.3. Schiff Base (SB) Content

SBs are formed through the reaction of carbonyl groups and amines, and can be produced when free amino groups in proteins combine with carbonyl groups from lipid-derived aldehydes. Thus, the SB content can to some extent represent the interaction between protein oxidation and lipid oxidation [20]. The effect of cooking on SB content is illustrated in Figure 1C. Compared with the R group, the BO and MW treatments exhibited a lesser impact on fluorescence intensity. On the other hand, the RS, SF and DF treatments significantly improved the fluorescence intensity, with DF treatment having the most pronounced effect (more than 10-fold higher than R). This indicated that higher heating temperatures and more intense cooking processes may aggravate protein oxidation and lipid oxidation in meat, leading to the production of more SBs. Similar findings were reported in [31]. As depicted in Figure S3 in the Supplementary Materials, both deep-fried and stir-fried chicken demonstrated diminished levels of lipid oxidation. This could be attributed to the potential interaction between malondialdehyde, a byproduct of lipid oxidation, and the free amino groups of the protein, resulting in the formation of SBs.

### 3.2. Impact of Various Cooking Methods on the Protein Structure of Chicken

#### 3.2.1. Protein Surface Hydrophobicity

**Figure 2:**
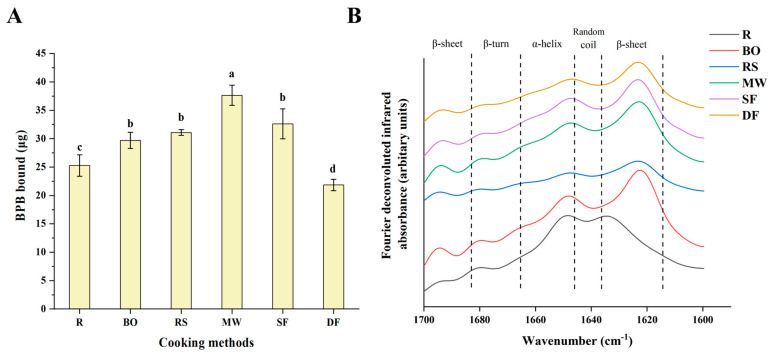
Effects of different cooking methods on the protein structure of chicken. (**A**) Surface hydrophobicity. (**B**) Fourier deconvoluted amide I (1700 to 1600 cm^−1^). R: uncooked raw meat. BO: boiling. RS: roasting. MW: microwaving. SF: stir-frying. DF: deep-frying. Different lowercase letters represent statistically significant differences between each group (*p* < 0.05).

Surface hydrophobicity can serve as one of the indicators to measure protein denaturation. Its increase is attributed to a change in protein conformation, specifically the unfolding of the protein structure and the exposure of non-polar amino acids on the protein surface [32]. It is generally believed that the degree of protein oxidation, particularly changes in the content of sulfhydryl groups, is associated with changes in protein conformation [33]. As depicted in Figure 2A, the four heat treatment methods of BO, RS, MW and SF all significantly increased the surface hydrophobicity of meat samples (*p* < 0.05). Interestingly, the RS and DF groups, which exhibited a higher degree of oxidation in Section 3.1, did not show a correspondingly higher surface hydrophobicity, and indeed the DF group (21.85 μg) exhibited lower surface hydrophobicity compared with the R group (25.27 μg) (*p* < 0.05). This suggests that protein oxidation is not the sole cause of structural changes. During the heat treatment process, the main reason for structural changes is the breakage of hydrogen bonds. If the heating is too intense, the exposed hydrophobic groups may be lost, resulting in a decrease in surface hydrophobicity. A similar phenomenon was also reported in [34]. These authors found that the surface hydrophobicity of the abalone fillet significantly increased after heat treatment but decreased with increasing heating time.

#### 3.2.2. Protein Secondary Structure

To examine the specific changes in the secondary structure, the deconvolution (Figure 2B) and second derivative peak fitting (Figure S4) were carried out in the amide I region. Table 1 demonstrates that in comparison to the R group, the relative proportions of α-helix and β-turn were significantly reduced in all cooking-treated samples, while the relative proportions of β-sheet were significantly increased (*p* < 0.05). This finding was consistent with a previous study [35]. This might be due to the fact that during heat treatment, a large number of hydrogen bonds, the main force that maintains the α-helix and β-turn, broke and formed a high proportion of β-sheet, among which the relative proportion of β-sheet in the SF and DF groups was relatively high, at 50.75% and 52.61%, respectively. Similarly, a previous study [36] reported that deep-frying significantly reduced the relative proportion of α-helix in peanut protein compared with boiling. Therefore, it could be speculated that cooking, especially when oil is added, might exhibit a greater impact on the secondary structure of proteins. Notably, the conformation of the β-sheet indicates protein aggregation to some extent, and so a higher proportion of β-sheet may affect the recognition sites of GI digestive enzymes, resulting in a decrease in the GI digestibility of proteins [37,38].

### 3.3. Impact of Various Cooking Methods on Digestive Characteristics of Chicken Protein

#### 3.3.1. Digestibility

**Figure 3:**
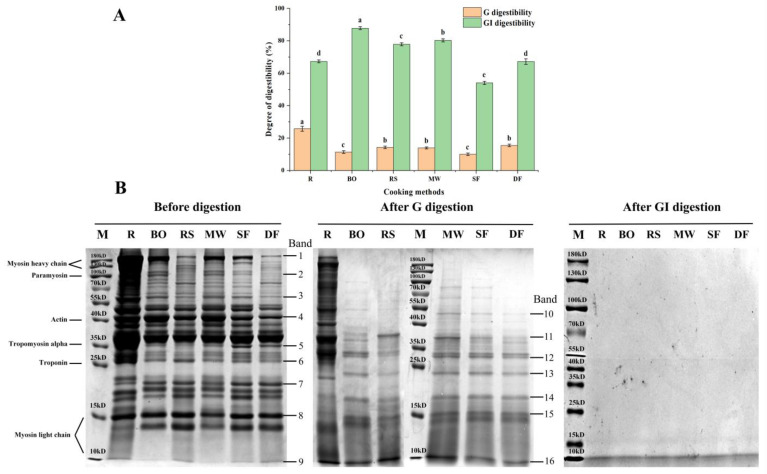
Digestion characteristics of chicken cooked with different methods. (**A**) Protein digestibility in vitro. (**B**) SDS-PAGE images of proteins before and after in vitro digestion. R: uncooked raw meat. BO: boiling. RS: roasting. MW: microwaving. SF: stir-frying. DF: deep-frying. M: molecular weight marker. G: gastric digestion. GI: gastrointestinal digestion. Different lowercase letters represent statistically significant differences between each group (*p* < 0.05).

Figure 3A shows the G digestibility and GI digestibility of meat samples after cooking treatment. After G digestion, the digestibility of cooked meat samples exhibited a significant decrease (*p* < 0.05) compared with the R group (25.74%). This finding aligned with a previous study [39]. This decrease in digestibility might be attributed to protein oxidation and structural changes during the cooking process, which disrupted the recognition site for pepsin. After GI digestion, the SF group showed the lowest digestibility (54.05%), followed by the DF (67.17%), RS (77.88%) and MW (80.27%) groups. The BO group exhibited the highest digestibility (87.77%). This finding aligned with a previous study [40], documenting that fried rabbit meat was more difficult to digest than boiled rabbit meat. Interestingly, in Section 3.1 and Section 3.2, the SF and DF groups exhibited a relatively higher degree of protein oxidation and structural changes. This might have destroyed the recognition site of trypsin, resulting in decreased digestibility. On the other hand, mild oxidation and structural changes, such as the BO and MW groups, partially unfolded the protein structure, exposed more recognition sites for binding to trypsin, and increased digestibility [41].

#### 3.3.2. SDS-PAGE Patterns

As seen in Figure 3B and Table 2, the relative intensities of band 1, 2, 3, 5 and 6 (25 kD to 180 kD) of the meat samples from all cooking treatments decreased significantly (*p* < 0.05) before digestion, indicating that cooking treatments easily decomposed high-molecular-weight proteins into smaller peptides or free amino acids. Furthermore, band 1 (180 kD), 2 (100 kD), and 3 (55 kD) indicated that the relative intensities of RS, SF, and DF groups were significantly weaker than the BO and MW groups. Among them, the DF group showed the lowest relative intensity (*p* < 0.05). In Section 3.1, the SF, RS, and DF groups exhibited higher degrees of protein oxidation, which might contribute to the degradation of high-molecular-weight protein structures such as myosin heavy chains and paramyosin. This was also consistent with the changes in protein secondary structure observed in Section 3.2.2. Previous studies have also reported similar phenomena [42,43], indicating that the band intensities of pork proteins with a high molecular weight decreased significantly with increasing cooking temperature. However, for low-molecular-weight band intensities of 6, 7, 8 and 9 (10 kD to 15 kD), no significant difference was observed between the SF, RS and DF groups, and some band intensities were even slightly stronger than the BO and MW groups (*p* > 0.05). It indicated that high-temperature treatment might degrade high-molecular-weight proteins into lower-molecular-weight proteins such as myosin light chains.

After G digestion, the relative intensities of all bands were significantly decreased, and a new band, 13, appeared, confirming the hydrolytic effect of pepsin on proteins. Bands 10, 13 and 16 indicated that the relative intensities of the RS, SF and DF groups remained weak (*p* < 0.05), following the same trend observed before digestion. This finding was similar to the results reported previously [44]. Bands 14 and 15 demonstrated that different cooking methods did not significantly affect their relative intensities, possibly due to the limited effect of pepsin on small molecular-weight proteins. Hardly any bands were visible after GI digestion, indicating that the trypsin hydrolysis of proteins was relatively thorough, which aligned with the findings reported previously [45].

#### 3.3.3. Microstructure

**Figure 4:**
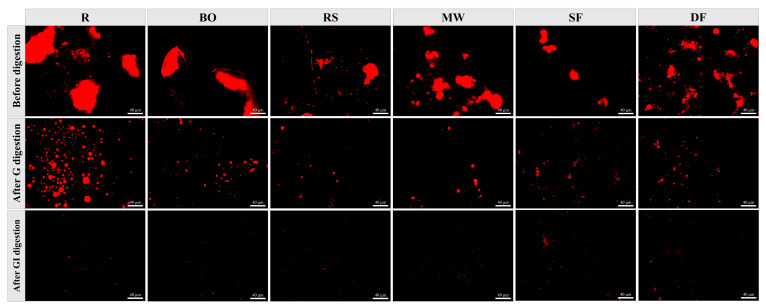
Fluorescence microscope images of chicken treated with different cooking methods before and after in vitro digestion. R: uncooked raw meat. BO: boiling. RS: roasting. MW: microwaving. SF: stir-frying. DF: deep-frying. G: gastric digestion. GI: gastrointestinal digestion.

Laser confocal microscopy was employed to observe the microstructural changes in chicken before and after digestion with different cooking methods. The bright red fluorescent spots observed in Figure 4 represented protein particles stained with Nile blue reagent. Before digestion, the sizes of protein particles decreased in all five cooking samples. The BO and MW groups showed only a slight decrease, while the RS, SF and DF groups exhibited significant reductions. This indicates that cooking treatments, especially those involving high temperatures and intense processing, led to the destruction of the protein structure of large particles, which aligned with the findings from SDS-PAGE. After G digestion, the particle size of all samples was notably decreased due to the action of pepsin, following a similar relative trend observed before digestion. After GI digestion, all protein particles were further decomposed into small peptides, which was consistent with the previous research [46]. However, in the SF and DF groups, some aggregated particles could still be observed, corresponding to the lower GI digestibility observed in these cooking methods.

### 3.4. Impact of Various Cooking Methods on the Colonic Fermentation Characteristics of Chicken

#### 3.4.1. Microbial Community

**Figure 5:**
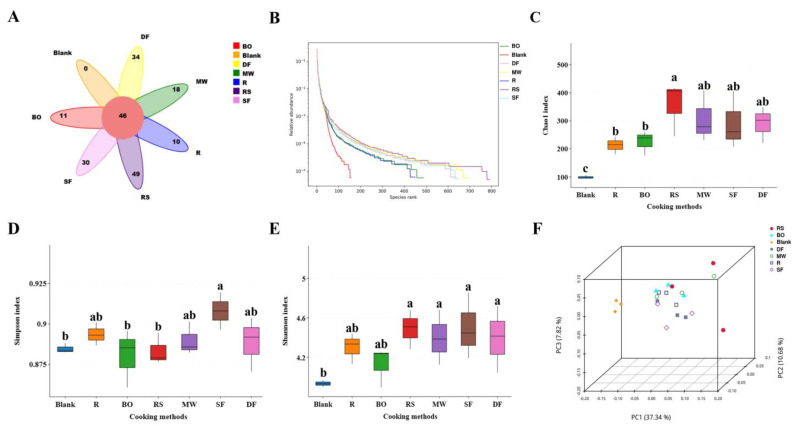
Effects of different cooking methods of chicken on intestinal flora diversity. (**A**) Species petal map. (**B**) Rank abundance curves. (**C**) Chao 1 index. (**D**) Simpson index. (**E**) Shannon index. (**F**) Principal coordinate analysis (PCoA) of the intestinal flora at the OTU level (unweighted-unifrac-full-tree distance). R: uncooked raw meat. BO: boiling. RS: roasting. MW: microwaving. SF: stir-frying. DF: deep-frying. Different lowercase letters represent statistically significant differences between each group (*p* < 0.05).

The diversity of the intestinal flora was assessed. In the species petal plot (Figure 5A), the RS group exhibited the highest number of new species (49) that were not found in the other cooking groups, indicating that the species richness of the RS group was the highest. At the same time, the rank abundance curve (Figure 5B) (alpha diversity) showed that the curves of the MW, RS, SF and DF groups all exhibited greater lengths compared with the blank group, indicating that they all improved the richness of intestinal flora species. The curve of the RS group was the longest, but an obvious step could be observed. This indicated that although the RS group exhibited the highest species richness, its species composition might have less evenness. This was further supported by the Chao 1 index (Figure 5C) and Shannon index (Figure 5D). The RS group exhibited the highest Chao 1 index (356.20), indicating the highest species richness [47], but its Simpson index (0.88) was relatively low, suggesting less evenness [48]. There was no significant difference in the Shannon index (Figure 5E) among the different cooking groups (*p* > 0.05). In addition, principal coordinate analysis (PCoA) (Figure 5F) demonstrated overall differences (beta diversity) in the intestinal flora. All meat samples were far away from the blank group, confirming the significant impact of protein fermentation on the intestinal flora. The RS group was farthest from the blank group, followed by the MW and DF groups. All of the results mentioned above indicated that RS treatment exhibited a significant effect on the alpha and beta diversity of intestinal flora.

**Figure 6:**
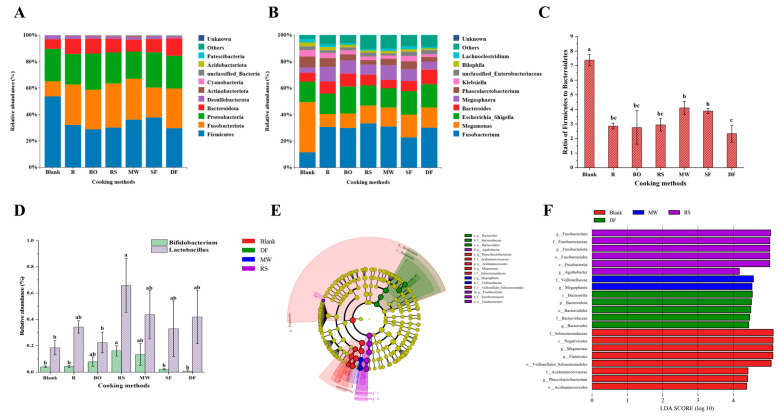
Effects of different cooking methods of chicken on intestinal flora composition. (**A**) Phylum abundance. (**B**) Genus abundance. (**C**) Ratio of *Firmicutes* to *Bacteroidota* (F/B). (**D**) Abundance of *Bifidobacterium* and *Lactobacillus*. (**E**) Linear discriminant analysis effect size (LEfSe) analysis. (**F**) Linear discriminant analysis (LDA). The LDA score was greater than 4. R: uncooked raw meat. BO: boiling. RS: roasting. MW: microwaving. SF: stir-frying. DF: deep-frying. Different lowercase letters represent statistically significant differences between each group (*p* < 0.05).

At the phylum level (Figure 6A), meat protein fermentation exhibited a noticeable effect on the flora structure compared with the blank group. The abundance of *Fusobacteriota* increased to varying degrees, with the RS group showing the highest increase (33.7%) and the SF group showing the lowest increase (22.8%). The abundance of *Proteobacteria* and *Bacteroidota* changed minimally, indicating that protein fermentation exhibited a limited effect on the growth of these phyla. It was worth noting that the *Firmicutes*-to-*Bacteroidota* ratio (F/B ratio), which is associated with chronic diseases such as obesity [49], was significantly decreased in all meat sample groups (Figure 6C). The MW and SF groups exhibited significantly higher F/B ratios compared with the other cooking groups, while DF group exhibited the lowest F/B ratio (*p* < 0.05). This might be related to *Bacteroidota* being a characteristic bacteria of the DF group, as indicated by the linear discriminant analysis effect size (LEfSe) analysis (Figure 6E,F).

At the genus level (Figure 6B), the abundances of *Fusobacterium* were all significantly increased (*p* < 0.05) after adding meat samples, suggesting that *Fusobacterium* might be the main genus involved in protein fermentation. It has been reported that the *Fusobacterium* is often enriched in colorectal cancer patients, so it may play a role in colon tumorigenesis [50]. Among all cooking groups, the RS group exhibited the highest increase in *Fusobacterium* abundance (33.33%). The Lefse analysis also showed that *Fusobacterium* was the characteristic bacteria of the RS group. Meanwhile, the abundance of *Megamonas*, as a probiotic [51], was significantly decreased in all groups with meat samples (*p* < 0.05). Additionally, *Bifidobacterium* and *Lactobacillus*, which are generally considered beneficial to human health [52], exhibited the highest abundance in RS group (0.16% and 0.66%, respectively) compared with other cooking groups (Figure 6D). The SF and DF groups showed lesser changes in these genera. All in all, when protein fermentation affects microbial diversity, an increase in the abundance of harmful bacteria and a decrease in the abundance of probiotics might also occur. This change is detrimental to health. Interestingly, it might also potentially increase the abundance of certain non-dominant probiotics.

It has been reported that an elevated transport of proteins to the large intestine might result in a decline in the abundance of intestinal probiotics, an increase in the abundance of harmful bacteria, and the generation of certain detrimental metabolites [53]. The findings of the current study suggest that the digestibility of the SF and DF groups was lower, that of the RS group was moderate, and the digestibility of the BO and MW groups was higher. Interestingly, the DF and SF groups (for which more protein reached the colon) did not exhibit a greater adverse effect on the intestinal flora than the RS group. As seen in Section 3.1.3, both the DF and SF groups produced more Schiff bases. Schiff bases can be formed through the combination of amino groups of proteins and lipid-derived aldehydes [20], or as an intermediate product of the Maillard reaction participating in Amadori rearrangement [54]. The oxidation of proteins and the formation of Maillard reaction products during thermal processing occur at the same time, and share the same precursors. The reaction pathways are related to each other [55]. Similarly, the previous studies reported that the enhancement of the Maillard reaction could reduce the intensity of protein colonic fermentation [10,56]. Therefore, we hypothesize that the addition of oil during cooking could attenuate the intensity of protein fermentation to some extent by enhancing the Maillard reaction.

#### 3.4.2. SCFA Analysis

**Figure 7:**
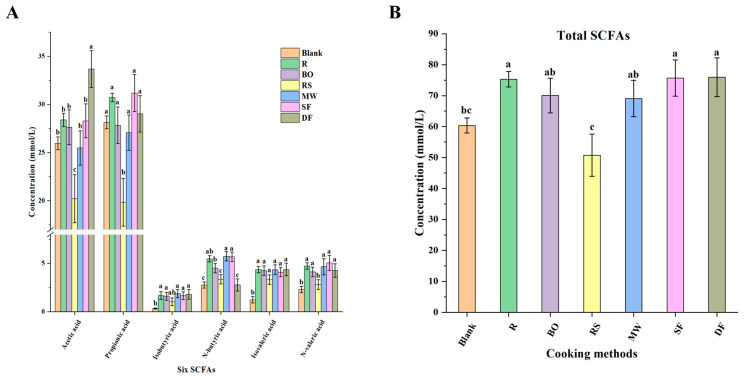
Effects of different cooking methods of chicken on the contents of six short-chain fatty acids (SCFAs) after colonic fermentation in vitro. (**A**) The contents of six SCFAs. (**B**) The contents of total SCFAs. R: uncooked raw meat. BO: boiling. RS: roasting. MW: microwaving. SF: stir-frying. DF: deep-frying. Different lowercase letters represent statistically significant difference between each group (*p* < 0.05).

The contents of six SCFAs and the total SCFAs were measured to evaluate the colonic fermentability of protein under different cooking methods. It has been reported that straight-chain short-chain fatty acids produced by anaerobic bacteria in the intestines through the breakdown of carbon sources not only provide energy for colon cells but also exert inhibitory effects on the growth of pathogenic bacteria [57]. In addition, they exhibited therapeutic efficacy in cellular cancer immunotherapy [58]. Therefore, they are generally thought to be beneficial for gut health. In Figure 7A, it is evident from the findings that acetic acid and propionic acid were the most abundant SCFAs (all between 20 and 35 mM) across all groups. Compared with other cooking groups, the RS group exhibited a significant decrease in four straight-chain short-chain fatty acids (*p* < 0.05). Equally, the total SCFA production (50.76 mM) in the RS group (Figure 7B) was also the lowest. This indicated that RS treatment exhibited an adverse effect on intestinal flora metabolism, which was consistent with the results of the diversity analysis presented in Section 3.4.1. The DF group produced the most acetic acid (33.68 mM) (*p* < 0.05), but its yield of n-butyric acid (2.78 mM) was lower (*p* < 0.05). This might be attributed to the presence of a large amount of oil, as the resulting Maillard reaction products exhibited a greater impact on acetogenic bacteria and butyric acid-producing bacteria. It is worth noting that isobutyric acid and isovaleric acid are branched-chain fatty acids, which are typically present in small proportions and are signature products of proteins’ anaerobic fermentation [59]. In the present research, the yields of isobutyric acid and isovaleric acid of all meat sample groups were significantly increased compared with the blank group (*p* < 0.05), with no significant difference among the meat sample groups. The process of protein fermentation was confirmed.

## 4. Conclusions

In the present study, all five cooking methods used—boiling (BO), roasting (RS), microwaving (MW), stir-frying (SF), and deep-frying (DF)—resulted in the oxidation of chicken proteins. The level of protein oxidation in the RS group was lower than that in the DF group but higher than in the other three cooking groups. The secondary structure of chicken proteins showed the most significant changes in the SF and DF groups. Digestibility, SDS-PAGE, and microstructure experiments all showed that the digestibility of the RS group was at a moderate level among all cooking groups. On the other hand, during colonic fermentation, the RS group exhibited the most adverse effect on the intestinal flora, while the SF and DF groups, despite their lower digestibility, did not demonstrate a significantly greater impact on intestinal flora compared with the BO and MW groups. Therefore, we conclude that cooking treatments, particularly those involving high temperatures and intense processes, could reduce the digestibility of chicken by intensifying protein oxidation and altering protein conformation. However, when digestibility was reduced and more protein reached the colon, the addition of oil during cooking might affect the intensity of the protein fermentation. The specific mechanism through which these cooking methods influence the intestinal flora is unclear, and thus further investigations to understand the underlying causes and possible mechanism of action are warranted. Anyway, roasting is the least recommended daily cooking method for chicken, and this is worth noting.

## Figures and Tables

**Table 1:** Relative proportions of the protein secondary structure of chicken with different cooking methods.

Cooking Methods	Secondary Structure Proportions (%)			
	α-Helix	β-Sheet	β-Turn	Random Coil
R	40.41 ± 0.24 ^a^	12.14 ± 0.17 ^e^	7.25 ± 0.18 ^a^	40.20 ± 0.26 ^a^
BO	35.41 ± 0.83 ^b^	43.57 ± 0.38 ^c^	3.39 ± 0.38 ^c^	17.63 ± 0.08 ^c^
RS	34.50 ± 0.85 ^b^	41.25 ± 0.17 ^d^	3.77 ± 0.50 ^c^	20.48 ± 1.41 ^c^
MW	35.30 ± 1.38 ^b^	42.27 ± 0.52 ^cd^	3.41 ± 0.33 ^c^	19.03 ± 1.19 ^c^
SF	14.11 ± 0.74 ^c^	50.75 ± 0.95 ^b^	5.17 ± 0.37 ^b^	29.97 ± 1.90 ^b^
DF	14.99 ± 0.19 ^c^	52.61 ± 0.33 ^a^	6.03 ± 0.06 ^b^	26.38 ± 0.09 ^b^

Values are shown as means ± SD (standard deviation) from triplicate determinations. R: uncooked raw meat. BO: boiling. RS: roasting. MW: microwaving. SF: stir-frying. DF: deep-frying. Different lowercase letters indicate statistically significant differences within a column (*p* < 0.05).

**Table 2:** SDS–PAGE relative band intensities of chicken protein before and after in vitro digestion under different cooking methods.

Bands Number	Relative Intensities					
	R	BO	RS	MW	SF	DF
Before digestion						
1	1.527 ± 0.079 ^a^	1.269 ± 0.059 ^b^	0.645 ± 0.027 ^d^	1.170 ± 0.054 ^b^	0.910 ± 0.027 ^c^	0.430 ± 0.019 ^e^
2	1.244 ± 0.179 ^a^	0.826 ± 0.105 ^b^	0.598 ± 0.083 ^bcd^	0.691 ± 0.092 ^bc^	0.429 ± 0.059 ^cd^	0.241 ± 0.022 ^d^
3	0.905 ± 0.054 ^a^	0.612 ± 0.038 ^b^	0.454 ± 0.030 ^c^	0.588 ± 0.038 ^b^	0.452 ± 0.025 ^c^	0.354 ± 0.020 ^c^
4	1.870 ± 0.045 ^a^	1.830 ± 0.071 ^a^	1.544 ± 0.076 ^bc^	1.746 ± 0.063 ^ab^	1.432 ± 0.057 ^cd^	1.264 ± 0.032 ^d^
5	0.952 ± 0.020 ^a^	0.543 ± 0.036 ^cd^	0.473 ± 0.034 ^d^	0.540 ± 0.035 ^cd^	0.699 ± 0.045 ^b^	0.634 ± 0.032 ^bc^
6	0.972 ± 0.080 ^a^	0.537 ± 0.030 ^b^	0.631 ± 0.032 ^b^	0.500 ± 0.030 ^b^	0.520 ± 0.022 ^b^	0.487 ± 0.023 ^b^
7	0.610 ± 0.032 ^b^	0.718 ± 0.029 ^ab^	0.717 ± 0.035 ^ab^	0.662 ± 0.034 ^ab^	0.730 ± 0.037 ^ab^	0.760 ± 0.030 ^a^
8	1.526 ± 0.028 ^a^	1.485 ± 0.021 ^ab^	1.471 ± 0.024 ^ab^	1.327 ± 0.024 ^c^	1.417 ± 0.026 ^bc^	1.418 ± 0.031 ^bc^
9	0.515 ± 0.052 ^a^	0.234 ± 0.021 ^b^	0.356 ± 0.047 ^b^	0.236 ± 0.015 ^b^	0.344 ± 0.015 ^b^	0.484 ± 0.036 ^a^
After G digestion						
10	0.711 ± 0.048 ^a^	0.154 ± 0.004 ^c^	0.158 ± 0.009 ^c^	0.281 ± 0.012 ^b^	0.176 ± 0.003 ^c^	0.053 ± 0.001 ^d^
11	0.732 ± 0.073 ^a^	0.299 ± 0.031 ^c^	0.469 ± 0.049 ^b^	0.535 ± 0.055 ^b^	0.299 ± 0.030 ^c^	0.184 ± 0.019 ^c^
12	0.733 ± 0.090 ^a^	0.460 ± 0.041 ^bc^	0.308 ± 0.013 ^c^	0.537 ± 0.055 ^b^	0.429 ± 0.037 ^bc^	0.341 ± 0.036 ^c^
13	0.401 ± 0.018 ^bc^	0.447 ± 0.017 ^ab^	0.309 ± 0.010 ^d^	0.475 ± 0.015 ^a^	0.391 ± 0.009 ^c^	0.318 ± 0.009 ^d^
14	0.495 ± 0.052 ^a^	0.574 ± 0.063 ^a^	0.486 ± 0.046 ^a^	0.590 ± 0.071 ^a^	0.487 ± 0.047 ^a^	0.436 ± 0.053 ^a^
15	0.588 ± 0.095 ^a^	0.697 ± 0.091 ^a^	0.643 ± 0.091 ^a^	0.867 ± 0.114 ^a^	0.750 ± 0.098 ^a^	0.703 ± 0.083 ^a^
16	1.131 ± 0.030 ^cd^	1.202 ± 0.019 ^bc^	1.288 ± 0.010 ^b^	1.520 ± 0.079 ^a^	1.037 ± 0.041 ^d^	0.875 ± 0.044 ^e^

Values are shown as means ± SD from triplicate determinations. R: uncooked raw meat. BO: boiling. RS: roasting. MW: microwaving. SF: stir-frying. DF: deep-frying. G: gastric digestion. Different lowercase letters indicate statistically significant differences within a row (*p* < 0.05).

## Data Availability

Data are contained within the article.

## Supplementary Materials

The following supporting information can be downloaded at: https://www.mdpi.com/article/10.3390/foods12234322/s1, Figure S1: Overview and flow diagram of a simulated in vitro digestion method; Figure S2: SHIME^®^ model of fermentation for 0, 4 and 8 days; Figure S3: Effects of different cooking methods on chicken lipid oxidation; Figure S4: Example of Gaussian multicomponent peak fitting model; Table S1: Specific procedure of the five cooking methods; Table S2: Specific components of the gastrointestinal simulated electrolyte solution; Table S3: Composition of the SHIME^®^ medium.
