# Supplementary material for: Cooking-Induced Oxidation and Structural Changes in Chicken Protein: Their Impact on In Vitro Gastrointestinal Digestion and Intestinal Flora Fermentation Characteristics
Source: Foods. 2023 Nov 29;12(23):4322. doi: 10.3390/foods12234322 (PMC10706749; doi:10.3390/foods12234322)
Supplement: Supplementary file 1 [file foods-12-04322-s001.zip › foods-2731844-supplementary.pdf]

Supplementary Materials

Cooking-Induced Oxidation and Structural Changes in Chicken Protein: Their Impact on In Vitro Gastrointestinal Digestion and Intestinal Flora Fermentation Characteristics

Guanhua Lv [1], Hengpeng Wang [1,2], Xiaoou Wei [1], Minmin Lu [1], Wenhao Yang [1], Halah Aalim [1], Esra Capanoglu [3], Xiaobo Zou [4,*], Maurizio Battino [5,6,*] and Di Zhang [1]

[1] School of Food and Biological Engineering, Jiangsu University, Zhenjiang 212013, China; 2212118055@stmail.ujs.edu.cn (G.L.); yzuwhp@163.com (H.W.); xiaoou_wei1997@163.com (X.W.); 2212118064@stmail.ujs.edu.cn (M.L.); ywh13615265085@163.com (W.Y.); a.halah@outlook.com (H.A.); d.zhang@ujs.edu.cn (D.Z.)

[2] Key Laboratory of Chinese Cuisine Intangible Cultural Heritage Technology Inheritance, Ministry of Culture and Tourism, College of Tourism and Culinary Science, Yangzhou University, Yangzhou 225127, China

[3] Department of Food Engineering, Faculty of Chemical and Metallurgical Engineering, Istanbul Technical University, 34469 Maslak, Türkiye; capanogl@itu.edu.tr

[4] International Joint Research Laboratory of Intelligent Agriculture and Agri-Products Processing, Jiangsu University, Zhenjiang 212013, China

[5] International Research Center for Food Nutrition and Safety, Jiangsu University, Zhenjiang 212013, China

[6] Department of Clinical Sciences, Faculty of Medicine, Polytechnic University of Marche, 60121 Ancona, Italy

* Correspondence: zou_xiaobo@ujs.edu.cn (X.Z.); m.a.battino@univpm.it (M.B.)

Table of content:

1. Supplementary experimental section Page S3-S6

2. Supplementary results and discussion Page S6-S7

3. Supplementary Figures (Figure S1-S4) Page S8-S11

4. Supplementary Tables (Table S1-S3) Page S12-S14

5. References Page S15

1. Supplementary experimental section

1.1 Materials and reagents

Arabinogalactan, Pectin, Xylan, Starch, Cysteine, Yeast extract, Glucose, Mucin, Peptone, 2-thiobarbituric acid (TBA), trichloroacetic acid (TCA) and 1,1,3,3-tetramethoxypropane (TEP) were purchased from Sigma-Aldrich (Shanghai, China).

1.2 Operational details of the SHIME® system

1.2.1 Fecal collection

The human gut microbes were collected from stool samples taken from six healthy adult males who had not been treated with antibiotics for six months. All volunteers signed an informed consent form before the experiment, and the study was approved by Ethics Committee of Jiangsu University. 70 g fresh fecal samples were added to 350 mL sterile phosphate buffered saline (0.1 M, pH=7.4), 1 g sodium thioglycolate was added as reducing agent, and thoroughly mixed to obtain 20% (w/v) fecal suspension, and finally centrifuged 1200 r/min for 10 min to obtain the supernatant.

1.2.2 *In vitro* colonic fermentation

In vitro gut fermentation used the SHIME system, referring to Neves Casarotti et al. for specific implementation methods [1]. The SHIME® system consisted of five glass containers with thermostatic water interlayers designed to simulate the stomach, small intestine, ascending colon, transverse colon and descending colon. The five containers were maintained at 37 °C through the constant temperature water bath device. Nitrogen

was passed every 8 h to maintain the anaerobic environment of each part of the reactor, and light avoidance measures was carried out. 750, 1200 and 900 mL of SHIME® nutrient medium were added into ascending, transverse and descending colon containers, respectively, and fecal supernatant were added. The pH of the ascending colon, transverse colon, and descending colon was controlled within the ranges of 5.5-5.9, 6.0-6.4, and 6.6-6.9, respectively, using a pH controller. 224 mL of SHIME® nutrient medium (Detailed ingredients are shown in Table S3) was added into the stomach container by peristaltic pump every 8 h at a speed of 7.8 mL/min for 28.7 min, and the medium remained in the stomach for 2 h. Two hours later, all the solution in the stomach was transferred to the small intestine container, and 112 mL pancreatic fluid was added into the small intestine by peristaltic pump at a speed of 7.2 mL/min for 15.6 min. The residence time of the mixture of medium and pancreatic fluid in the small intestine stage was 4 h, after which it was pumped into the ascending colon container. The three containers, including the ascending colon, transverse colon, and descending colon, were continuously flowed at a rate of 0.625 mL/min, with respective residence times of 20 h, 32 h, and 24 h. The above operational steps were carried out continuously for one week to ensure stable microbial growth and successful colonization.

Refer to the method of Yang et al. [2]. 0.1 g of freeze-dried intestinal digestive powder was mixed with 20 mL SHIME® culture solution, followed by anaerobic fermentation for 24 h and centrifugation for 5 min at 12000 r/min. The precipitation

was used for the detection of bacterial community structure, and the supernatant was used for the detection of short-chain fatty acids.

1.3 Quantification of thiobarbituric acid reactive substances (TBARS)

The values of TBARS were determined by the method of Jiang et al. [3]. The 3 mL supernatant was filtered after homogenizing chicken and 10% TCA. The 3 mL 0.02 M TBARS solution was added. Reaction was carried out in boiling water bath at 100 ℃ for 40 min. Absorbance was measured at 532 nm wavelength. A standard curve was made using TEP.

1.4 Microbial community analysis of 16S rRNA gene sequencing

Referring to the method of Yang et al. [2], total genomic DNA was extracted from the samples after fermentation for 24 h using the TGuide S96 Magnetic Soil / Stool DNA Kit (Tiangen Biotech (Beijing) Co., Ltd.). The hypervariable region V3-V4 of the bacterial 16S rRNA gene were amplified with primer pairs 338F: 5'-ACTCCTACGGGAGGCAGCA-3' and 806R: 5'- GGACTACHVGGGTWTCTAAT-3'. PCR products were checked on agarose gel and purified through the Omega DNA purification kit (Omega Inc., Norcross, GA, USA). The purified PCR products were collected and the paired ends (2 × 250 bp) was performed on the Illumina Novaseq 6000 platform.

The qualified sequences with more than 97% similarity thresholds were allocated to one operational taxonomic unit (OTU) using USEARCH (version 10.0). Taxonomy

annotation of the OTUs / ASVs was performed based on the Naive Bayes classifier in QIIME2 using the SILVA database with a confidence threshold of 70%.

2. Supplementary results and discussion

2.1 Impact of various cooking methods on the degree of chicken lipid oxidation

As seen in Figure S3, the BO, RS, and MW groups (1.0 to 2.0 mg/kg) showed significantly higher levels of lipid oxidation ($p < 0.05$) compared with the R group (0.65 mg/kg). However, the TBARS of SF and DF groups (less than 1.0 mg/kg) was lower than that of other cooking methods ($p < 0.05$). This phenomenon might be attributed to the continuous degradation of secondary oxidation products, such as aldehydes, into other volatile substances under intense cooking conditions. On the other hand, this could also be explained by the possibility that malondialdehyde reacted with the amino groups of proteins to form more SB. As described in Section 3.1.3, the meat samples after SF and DF resulted in higher production of SB. Similar findings were reported by Hu et al. [4].

2.2 Stability of the SHIME® system

In all the *In vitro* models, SHIME® is a simulation system that can dynamically simulate the digestive process in five parts of the human stomach, small intestine, and ascending, transverse, and descending colon. Compared with other *In vitro* simulation methods, SHIME® has unique advantages in dynamically simulating different parts of

the colon and studying the effects and changes of intestinal microbes. During the initial phase of the SHIME® system, a one-week training period using SHIME® base medium was conducted to facilitate the adaptation of inoculated gut microbes to the nutritional and physiological conditions within distinct colonic compartments. This process aimed to simulate the microbial community structure found in various regions of the human colon. In this study, it can be seen that after 4 and 8 days of continuous culture, the flora structure tended to be stable at phyla and family levels (Figure S2A and Figure S2B). The phyla level included *Firmicutes*, *Bacteroidetes*, *Proteobacteria* and *Actinobacteria*. The family level included *Enterobacteriaceae*, *Bacteroideaceae* and *Fusobacteriaceae*. They were all common human dominant intestinal flora. At the same time, the contents of six short-chain fatty acids, the characteristic metabolites of the flora, increased significantly and the relative proportion tended to be stable (Figure S2C), which indicated that the growth of intestinal flora had stabilized.

3. Supplementary Figures (Figure S1-S4)

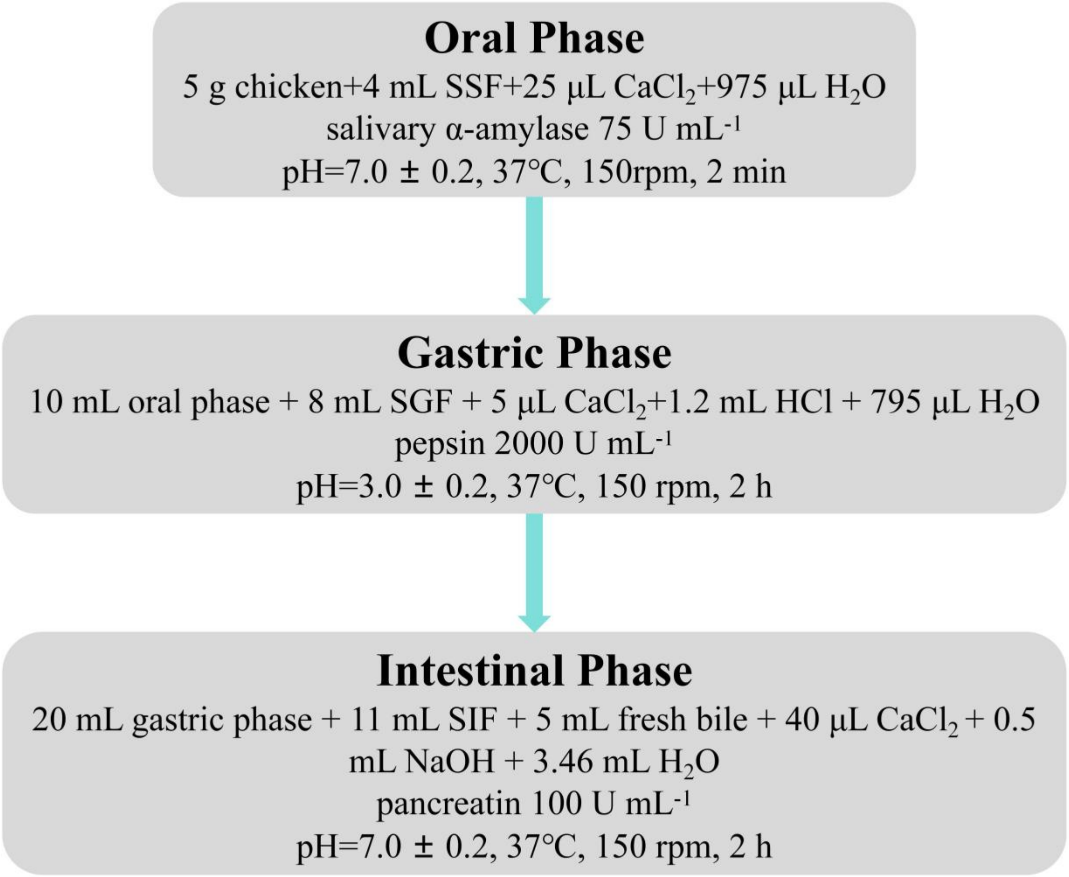

Figure S1. Overview and flow diagram of a simulated *in vitro* digestion method.

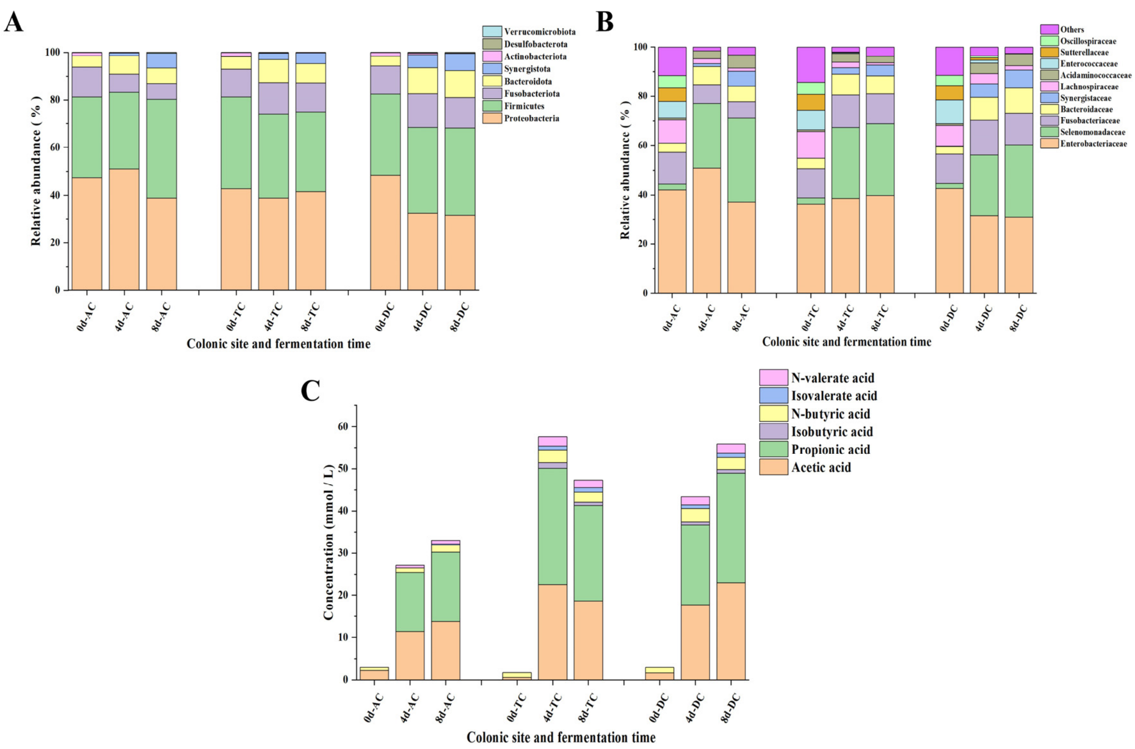

Figure S2. SHIME® model of fermentation for 0, 4 and 8 days. (A) Phylum abundance. (B) Family abundance. (C) Stack diagram of concentration of six SCFAs. AC: Ascending colon. TC: Transverse colon. DC: Descend colon.

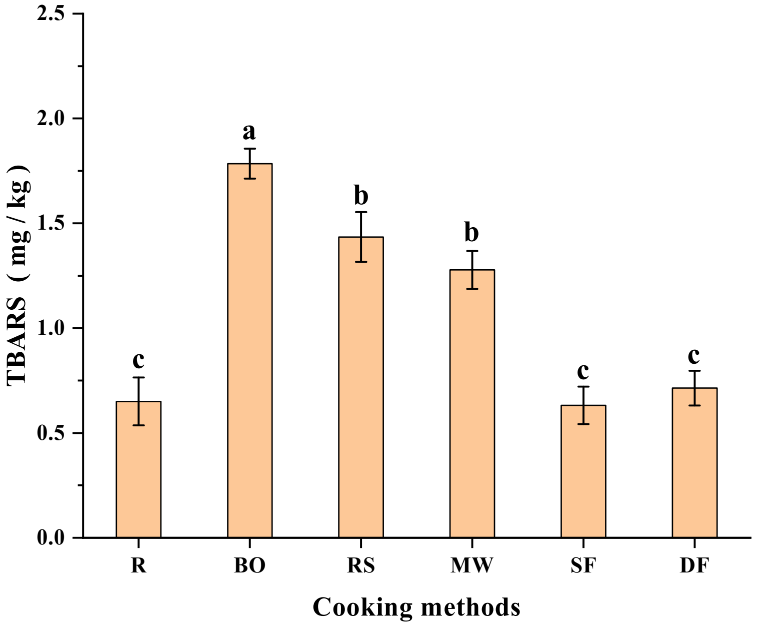

Figure S3. Effects of different cooking methods on chicken lipid oxidation (TBARS). Different lowercase letters represent statistically significant difference between each group ($p < 0.05$).

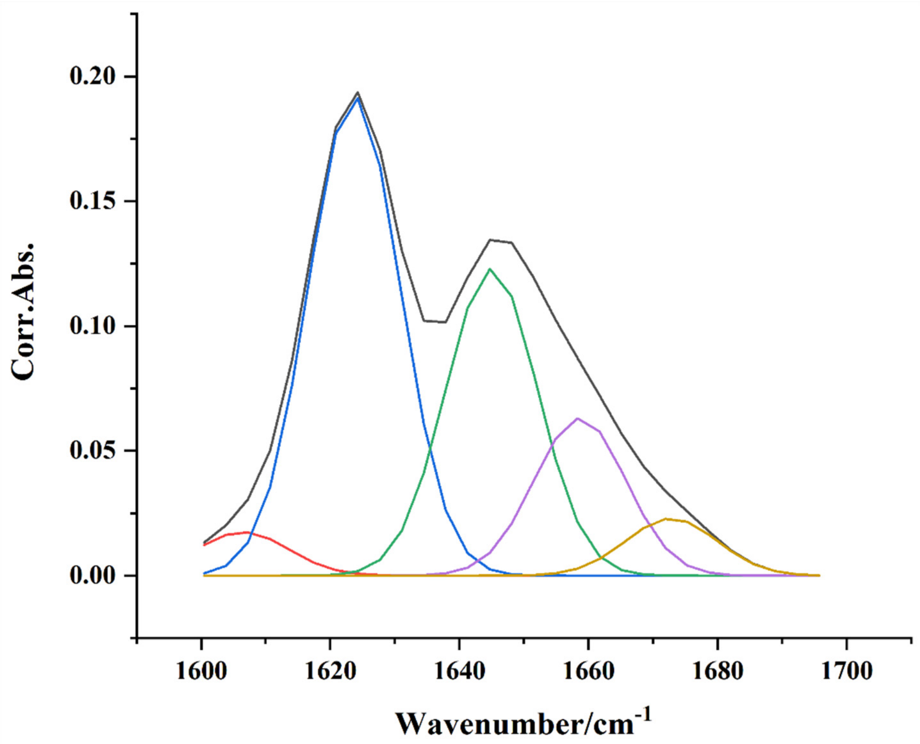

Figure S4. Example of Gaussian multicomponent peak fitting model (R^2 = 0.99).

4. Supplementary Tables (Table S1-S3)

Table S1. Specific procedure of the five cooking methods.

Cooking methods	Specific procedure
Boiling（BO）	Put the chicken pieces in boiling water for 3 min.
Roasting (RS)	Put the chicken pieces evenly into an electric oven for roasting. The temperature was set to 190 ℃. After roasting for 15 min, remove and turn them over, and roast for another 10 min.
Microwaving (MW)	Put the chicken in a porcelain bowl and microwave heating for 1.5 min.
Stir-frying (SF)	Soak a small amount of oil in the bottom of the pan. When the oil comes to a boil, add the chicken pieces and stir-fry for 3 min.
Deep-frying (DF)	Immerse the chicken pieces in oil pre-heated to 160 ℃ and fry for 1.5 min.

All processing group samples were in triplicate. All cooking methods end with the center temperature of the sample reaching about 80 ~ 90 ℃. No additional condiments were added during the cooking process. After cooling, the samples were ground and packed in vacuum bags (pieces: 200 × 270 × 0.090 mm^3) in the refrigerator at −80 ℃ for further testing.

Table S2. Specific components of the gastrointestinal simulated electrolyte solution.

Ingredient	SSF (mmol•L^{-1})	SGF (mmol•L^{-1})	SIF (mmol•L^{-1})
KCl	15.1	6.9	6.8
KH_2PO_4	3.7	0.9	0.8
$NaHCO_3$	13.6	25	85
NaCl	—	47.2	38.4
$MgCl_2(H_2O)$	0.15	0.1	0.33
$(NH_4)_2CO_3$	0.06	0.5	—
$CaCl_2(H_2O)_2$	1.5	0.15	0.6

Table S3 Composition of SHIME® medium.

Ingredient	Concentration （g/L）	Reagent	Concentration （g/L）
Arabinogalactan.	1.0	Yeast extract	1.0
Pectin	2.0	Glucose	0.4
Xylan.	1.0	Mucin.	4.0
Starch	4.0	Peptone	1.0
Cysteine	0.5		

References

1. Neves Casarotti, S.; Fernanda Borgonovi, T.; de Mello Tieghi, T.; Sivieri, K.; Lúcia Barretto Penna, A. Probiotic low-fat fermented goat milk with passion fruit by-product: In vitro effect on obese individuals' microbiota and on metabolites production. *Food Research International* **2020**, *136*, 109453. [https://doi.org/10.1016/j.foodres.2020.109453]
2. Yang, Y.; Wu, H.; Dong, S.; Jin, W.; Han, K.; Ren, Y.; Zeng, M. Glycation of fish protein impacts its fermentation metabolites and gut microbiota during in vitro human colonic fermentation. *Food Research International* **2018**, *113*, 189-196. [https://doi.org/10.1016/j.foodres.2018.07.015]
3. Jiang, S.; Feng, X.; Zhang, F.; Wang, R.; Zeng, M. Effects of cooking methods on the Maillard reaction products, digestibility, and mineral bioaccessibility of Pacific oysters (Crassostrea gigas). *LWT* **2021**, *141*, 110943. [https://doi.org/10.1016/j.lwt.2021.110943]
4. Hu, L.; Ren, S.; Shen, Q.; Chen, J.; Ye, X.; Ling, J. Proteomic study of the effect of different cooking methods on protein oxidation in fish fillets. *RSC Advances* **2017**, *7*, 27496-27505. [https://doi.org/10.1039/C7RA03408C]
